# Consumption of fruit and vegetables and the risk of type 2 diabetes: a 4-year longitudinal study among Swedish adults

**DOI:** 10.1017/jns.2020.7

**Published:** 2020-04-02

**Authors:** Arif Ahmed, Anton Lager, Peeter Fredlund, Liselotte Schäfer Elinder

**Affiliations:** 1 Department of Public Health Sciences, Stockholm University, Stockholm, Sweden; 2 Department of Demography, Stockholm University, Stockholm, Sweden; 3 Centre for Epidemiology and Community Medicine, Stockholm Region, Stockholm, Sweden; 4 Department of Public Health Sciences, Karolinska Institutet, Stockholm, Sweden

**Keywords:** Diet, Chronic diseases, Longitudinal studies, Sex differences, MET, metabolic equivalent of task, T2D, type 2 diabetes

## Abstract

A low intake of fruit and vegetables is a significant contributor to the global burden of disease. The aim of this study was to estimate the size of the risk of type 2 diabetes (T2D) of a low intake and to investigate possible sex differences. In this regard, this study used a longitudinal data from the Stockholm Public Health Cohort located in Sweden, collected in 2010 and 2014. The analysis included 14 718 men and 20 589 women aged 25 to 84 years. Fruit and vegetable intake, separately <2 servings/d or combined <4 servings/d (one serving corresponding to 100 g) was set as a cut-point for low intake. The sex difference at baseline was examined. Sex-stratified logistic regression was performed with onset of T2D as the outcome and fruit and vegetable intake at baseline as the exposure with adjustment for other known risk factors. Results indicate that men consumed significantly (*P* < 0⋅001) less fruit and vegetables compared with women. A 62 % higher risk to develop T2D over the 4-year period was observed in men who had low vegetable intake compared with high intake after adjusting for age, education, BMI, smoking, alcohol and physical activity (OR 1⋅62; 95 % CI 1⋅00, 2⋅63). In women, a significantly higher risk of T2D was also observed with a low intake of vegetables, but not after adjustment. The present study suggests that higher consumption of vegetables seems to be protective for the onset of T2D in men. Thus, increasing the intake of vegetables in men should be a public health priority.

Type 2 diabetes (T2D) belongs to the top ten causes of premature death, accounting for 90 % of all diabetes cases worldwide^(1)^. The prevalence of T2D was one in eleven adults, meaning 425 million, worldwide in 2017, and accounted for 12 % of the world's healthcare costs. Moreover, a rise to 629 million by 2045 in adults aged 20–79 years has been projected. In Sweden, the T2D prevalence is expected to rise from 0⋅5 million in 2017 to 0⋅6 million by 2045 in this age group^(1)^, and prevention efforts are therefore called for. T2D is a chronic metabolic disease caused by a combined effect of behavioural factors such as dietary habits and physical activity, and genetic and epigenetic factors^(2,3)^. In T2D, the body fails to produce sufficient insulin or becomes resistant to insulin, or both, which results in hyperglycaemia^(1,2)^.

Risk factors of T2D other than diet include family history of diabetes, overweight and obesity, higher age, low education, smoking, low physical activity and country of birth^(4,5)^. In all age groups in Sweden, T2D is more prevalent in men than in women^(6)^ and there is a steep social gradient to the disadvantage of people with low socio-economic status^(7)^.

About 10 % of the disease burden globally is estimated to be caused by unhealthy dietary habits^(6)^ causing diabetes, CVD and certain cancers. A low intake of fruit and vegetables is among the top five dietary risk factors for chronic diseases together with a low intake of whole grains, high intake of salt and low intake of nuts and seeds. Fruit, berries and vegetables are rich in fibre, K, antioxidants, folate^(8)^, minerals, vitamins, bioactive phytochemicals, carotenoids^(9)^ and polyphenolic compounds^(10)^ which may have beneficial effects on glucose metabolism. Furthermore, fruit and vegetables are rich in dietary fibre, which protects against weight gain^(11)^ and high insulin levels^(12)^. Reviews on the importance of dietary factors in the aetiology of CVD and diabetes have identified ten foods with possible cardiometabolic effects including protective effects of fruits, vegetables, beans/legumes, nuts/seeds, whole grains, fish, and yoghurt; and harmful effects of unprocessed red meats, processed meats and sugar-sweetened beverages^(13)^. Wu *et al*.^(14)^ performed a dose–response meta-analysis of prospective cohort studies and found that two to three servings/d of vegetables and two servings/d of fruit conferred the lowest risk of T2D. A similar conclusion was drawn by Schwingshackl *et al*.^(15)^ in their meta-analysis. Dietary recommendations concerning fruit and vegetables vary between countries. For example, ≥400 g/d are recommended by the WHO and in England, ≥500 g/d in Sweden, ≥600 g/d in Denmark, ≥650–750 g/d in Norway, and 640–800 g/d in the USA^(16)^. One serving is approximately equivalent to 100 g.

According to the nationally representative Riksmaten study conducted over the period May 2010–July 2011, the intake of fruits and vegetables in Sweden remains too low. The mean intake level of fruits and berries was 147 and 105 g/d in adult women and men, respectively. Vegetable intake including pulses and roots but excluding potatoes was 182 and 169 g/d in women and men, respectively. The average intake of fruits, berries (including maximum of 100 ml of juice) and vegetables in women and men was 360 g/d (72 % of recommended intake) and 310 g/d (62 % of recommended intake), respectively, with 21 % eating more than 500 g/d^(17)^.

A study on adults from Stockholm County observed an increasing prevalence of T2D (from 2⋅8 % in 1990 to 4⋅6 % in 2010) and incidence (from 2⋅6 per 1000 individuals in 1990 to 4⋅6 per 1000 individuals in 2010), with a higher incidence among men than women^(18)^. Hereafter the incidence has been around 4⋅4 per 1000 individuals in Stockholm^(19)^. Along with increasing global prevalence and incidence of diabetes, a doubling of deaths from diabetes during the period 2005 to 2030 has been projected by the WHO^(20)^. Death and disability in diabetics is mainly due to CVD^(21)^. According to the WHO, 80 % of T2D is preventable^(22)^. Effective strategies for the prevention of T2D are physical activity and a healthy diet including a high intake of fruit and vegetables, maintaining a normal body weight and avoiding smoking^(3,23)^.

The aims of the present study were to: (a) describe the mean baseline consumption of fruit and vegetables in Stockholm County in 2010 in men and women in relation to age, education and BMI; (b) describe the change in mean consumption of fruit and vegetables from 2010 to 2014; and (c) analyse a possible association between the level of consumption of fruit and vegetables and the onset of T2D over a 4-year period in men and women, respectively.

## Methods

### Ethical consideration

This study was conducted according to the guidelines laid down in the Declaration of Helsinki. Confidentiality of personal information is protected by means of pseudonymisation so that Statistics Sweden has no access to survey data and the Centre for Epidemiology and Community Medicine has no access to information on personal identity. The burden of the subjects is limited to filling in the questionnaire. This study was conducted within the scope of the responsibility of the Centre for Epidemiology and Community Medicine to generate knowledge on public health, disease prevention and health care. Statistical work conducted for these purposes at the Swedish regions and other public authorities are not contingent on ethical research board reviews. All subjects were informed in writing about the purposes with, and the procedures of, the survey and subsequently consented by means of returning the filled-in questionnaire.

### Data material

Data were from the Stockholm Public Health Cohort (SPHC), an area-stratified random sample of adults aged 18–84 years. The cohort and the sampling procedure have been described by Svensson *et al*.^(24)^. In comparison with Stockholm County census data, cohort members were somewhat more likely to be female, aged 45 years or older, born in Sweden and to have higher education and income. Data used for this study were collected by postal or web-based questionnaire in 2010 (baseline), with a response rate of 56 % and again in 2014 with a response rate of 71 % at follow-up. There were 49 421 respondents who provided information both in 2010 and in 2014.

Respondents below 25 years in 2010 were excluded from the analysis, as the few new cases occurring over the follow-up period would probably include a high proportion of type 1 diabetes. We also excluded individuals with pre-existing disease at baseline such as angina and heart failure which could have affected their health-related behaviours. The analytical sample therefore included respondents aged 25 to 84 years with complete information for all the studied variables and free from self-reported chronic diseases at baseline (2010). Thus, the analysis included 35 307 individuals (14 718 men and 20 589 women).

### Assessment of outcome: onset of type 2 diabetes

Diabetes in 2010 and 2014 was assessed with the question ‘Have you received any diagnoses for diabetes from a doctor?’ – ‘yes’ or ‘no’. T2D new cases were identified from the respondents free of T2D at baseline but reporting to have a diabetes diagnosis in 2014.

### Assessment of exposures

Three exposures were studied, namely intake of fruit, vegetables, and combined fruit and vegetables in 2010 and 2014. To assess the consumption of (a) fruit and (b) vegetables, the respondents were asked to estimate how many servings (per month, per week, per d) they consumed over the previous 12 months. The term fruit included fruit and berries (fresh, frozen, conserves, juices, etc.) and ‘vegetables’ referred to vegetables, leguminous plants, root vegetables (fresh, frozen, conserves, in sauces, etc. – but not potatoes). Fruit intake and vegetable intake were categorised into ‘<2 servings/d’ and ‘≥2 servings/d’, respectively, and named ‘low intake’ and ‘high intake’. The variable combined fruit and vegetable intake was created from the information collected for fruit and vegetable consumption and categorised into ‘<4 servings/d’ and ‘≥4 servings/d’ and named as ‘low intake’ and ‘high intake’.

### Assessment of other risk factors

Other established risk factors for T2D included in the analysis were age, education, country of birth, BMI, smoking, alcohol, and physical activity in 2010. A brief description of creation and categorisation of these variables is given below. Age was categorised into three groups (‘25–44’, ‘45–64’ and ‘65–84’ years) but used as a continuous variable in binary logistic regression analysis. BMI was created by using the self-reported height and weight of the respondents with the formula BMI = weight (kg)/height (m^2^). BMI was categorised into ‘BMI<25⋅0 kg/m^2^’, ‘overweight (25⋅0 ≤ BMI<30⋅0 kg/m^2^)’ and ‘obese (BMI ≥ 30⋅0 kg/m^2^)’ according to the WHO cut-off points^(25)^. Respondents with BMI less than 15⋅0 and higher than 50⋅0 kg/m^2^ were excluded to avoid suspected misreporting. Education was recoded as ‘basic education’ corresponding to 9 years or fewer, ‘secondary education’ corresponding to 10–12 years of schooling and ‘college or university education’ – named as ‘higher education’. Alcohol consumption was categorised into ‘no’, ‘low’, ‘medium’ and ‘high’ with different cut-off points for men and women, and those were ‘0’, ‘>0 to ≤24 g/d’, ‘>24 to ≤60 g/d’ and ‘>60 g/d’ for men; and ‘0’, ‘0 to ≤15⋅4 g/d’, ‘>15⋅4 to ≤50 g/d’ and ‘> 50 g/d’ for women, respectively^(5)^. Smoking status was created with the variables ever smoked (‘Have you ever smoked as good as daily, for at least 6 months?’ Answer with ‘yes’ or ‘no’) and current smoking (‘Are you currently smoking daily?’ Answer with ‘yes’ or ‘no’) and categorised into ‘never smoked’, ‘current smoker’ and ‘former smoker’^(5)^. Physical activity during the last 12 months was assessed through a combination of the following variables: walking/cycling, exercise, daily activities and/or work, domestic tasks, and sedentary activities. The variable ‘MET min/week’ was calculated by multiplying the metabolic equivalent of task (MET) value for each activity by time. Next, using the values MET min/week, the individuals were ranked and grouped into tertiles ‘low’, ‘medium’ and ‘high’ physical activity. Country of birth was grouped into ‘Sweden’ or ‘other’. To facilitate the comparison of the results for men and women, the same covariates were used in the analyses for men and women.

### Statistical analysis

All analyses were stratified by sex. The descriptive part includes number and percentage of respondents in each category of the variables. In order to test differences in intake of fruit and vegetables according to sex, age, educational level and BMI, the Mann–Whitney *U* test and the Kruskal–Wallis test were used. In order to test changes in intake over time, paired-sample *t* tests were used. Multivariate logistic regression was used to explore the associations between exposure in 2010 and the binary outcome for onset of T2D^(26,27)^. Five models were used: an unadjusted model, model 1; model 2 adjusted for weight status (BMI); model 3 adjusted for age and education; model 4 adjusted for age, education and weight status; and model 5 adjusted for the confounders used in model 4 and in addition smoking, alcohol and physical activity. The reference group consisted of individuals who had the lowest level of risk (high intake of fruit and vegetables) and the other groups were compared with that reference group^(28)^. Before using binary logistic regression, data were checked for multi-collinearity among the explanatory variables. No multi-collinearity was found as we received variance inflation factor (VIF) <5 for all the independent variables^(29)^.

#### Goodness-of-fit test

Hosmer–Lemeshow's test and area under the receiver operating characteristic (ROC) curve were used to assess whether the estimated model predicted the outcome better compared with the unadjusted model^(30,31)^.

#### Sensitivity analysis

A sensitivity analysis was done comparing individuals who lacked information on one or several covariates with those with complete data. For that, logistic regression was used to examine the association between the exposure and the outcome without adjustment for covariates except for age, since age was a strong confounder and had no missing values.

OR with 95 % CI were calculated. The level of significance was set at *P* < 0⋅05 All analyses were performed using IBM SPSS Statistics version 23^(32)^.

## Results

### Description of the cohort

Descriptive statistics of the cohort are shown in Table 1. Almost half (45⋅6 %) of the respondents were in the age range 45–64 years, and the mean age was 52⋅5 (sd 13⋅8) years. Women (54⋅3 %) had a slightly higher level of education than men (51⋅0 %). Almost half (44⋅8 %) of the sample had overweight or obesity (BMI ≥ 25 kg/m^2^), more men (55⋅1 %) than women (37⋅5 %), while 11⋅6 % of the respondents reported that they did not consume any alcohol. Current or former smoking was reported by 44⋅0 % of the participants. In total, 319 (167 in men and 152 in women) incident cases of T2D were observed during the 4-year follow-up period. The cumulative incidence of T2D was nine per 1000 in this sample over the 4-year period, higher among men (2⋅75 per 1000 per year) than women (2⋅25 per 1000 per year) (Table 1).

**Table 1.** Descriptive statistics of the studied variables in the total sample (*n* 35 307) and stratified for men (*n* 14 718) and women (*n* 20 589) at baseline (2010) and follow-up (2014) (Numbers and percentages)

Variables	2010						2014					
	Total (*n* 35 307)	%	Men (*n* 14 718)	%	Women (*n* 20 589)	%	Total (*n* 35 307)	%	Men (*n* 14 718)	%	Women (*n* 20 589)	%
Vegetable intake												
<2 servings/d	24 515	69⋅4	11 629	79⋅0	12 886	62⋅6	25 721	72⋅8	12 012	81⋅6	13 709	66⋅6
≥2 servings/d	10 792	30⋅6	3089	21⋅0	7703	37⋅4	9586	27⋅2	2706	18⋅4	6880	34⋅4
Fruit intake												
<2 servings/d	24 505	69⋅4	12 094	82⋅2	12 411	60⋅3	26 550	75⋅2	12 537	85⋅2	14 013	68⋅1
≥2 servings/d	10 802	30⋅6	2624	17⋅8	8178	39⋅7	8757	24⋅8	2181	14⋅8	6576	31⋅9
F&V intake												
<4 servings/d	28 123	79⋅7	13 209	89⋅7	14 914	72⋅4	29 746	84⋅2	13 496	91⋅7	16 250	78⋅9
≥4 servings/d	7184	20⋅3	1509	10⋅3	5675	27⋅6	5561	15⋅8	1222	8⋅3	4339	21⋅1
Country of birth												
Sweden	30 958	87⋅7	13 021	88⋅5	17 937	87⋅1						
Others	4349	12⋅3	1697	11⋅5	2652	12⋅9						
Age (years)												
25–44	11 168	31⋅6	4334	29⋅4	6834	33⋅2						
45–64	16 115	45⋅6	6751	45⋅9	936	45⋅5						
65–84	8024	22⋅7	3633	24⋅7	4391	21⋅3						
Education level												
Basic	3325	9⋅4	1536	10⋅4	1789	8⋅7						
Secondary	13 296	37⋅7	5688	38⋅6	7608	37⋅0						
Higher	18 686	52⋅9	7494	51⋅0	11 192	54⋅3						
BMI (kg/m^2^)												
BMI <25⋅0	19 489	55⋅2	6614	44⋅9	12 875	62⋅5						
25⋅0 ≤ BMI<30⋅0	12 300	34⋅8	6586	44⋅7	5714	27⋅8						
BMI ≥30⋅0	3518	10⋅0	1518	10⋅4	2000	9⋅7						
Alcohol intake (g/d)												
No	4095	11⋅6	1375	9⋅3	2720	13⋅2						
Low	21 233	60⋅1	9007	61⋅2	12 226	59⋅4						
Medium	9362	26⋅5	3897	26⋅5	5465	26⋅5						
High	617	1⋅8	439	3⋅0	178	0⋅9						
Smoking												
Never smoked	19 797	56⋅0	8422	57⋅2	11 375	55⋅2						
Current smoker	3272	9⋅3	1203	8⋅2	2069	10⋅0						
Former smoker	12 238	34⋅7	5093	34⋅6	7145	34⋅8						
Physical activity												
Low	12 085	34⋅2	5406	36⋅7	7034	34⋅2						
Medium	11 972	33⋅9	4891	33⋅2	6942	33⋅7						
High	11 250	31⋅9	4421	30⋅1	6613	32⋅1						
T2D new cases												
No							34 988	99⋅1	14 551	98⋅9	20 437	99⋅3
Yes							319	0⋅9	167	1⋅1	152	0⋅7

*n*, Number of respondents with valid data; F&V, fruit and vegetables; T2D, type 2 diabetes.

### Consumption of fruit and vegetables

Almost three-quarters of the respondents consumed fewer than two servings of vegetables per d. The proportion of men and women who consumed fewer than two servings of vegetables per d was 79⋅0 % at baseline and 62⋅6 % at follow-up. Similar results were found for fruit intake for the total sample as well as for men and women separately. Only 20⋅3 % of the individuals consumed four or more servings per d, with about 3-fold difference between men (10⋅3 %) and women (27⋅6 %), and slightly lower proportions at follow-up (Table 1).

The overall mean consumption of fruit was 1⋅2 (sd 0⋅8) portions/d, vegetables 1⋅2 (sd 0⋅9) portions/d, and combined fruit and vegetables 2⋅4 (sd 1⋅4) portions/d. A significantly (*P* < 0⋅001) lower intake of fruit and vegetables was observed in men compared with women at baseline. The older age group had the lowest consumption of vegetables, while fruit consumption was lowest among the youngest age group. Similarly, low-educated and obese individuals had the lowest consumption of fruit and vegetables (Table 2).

**Table 2.** Population characteristics based on data for 2010 by mean consumption of fruits, vegetables and fruit and vegetables (F&V) in Swedish adults (*n* 35 307) (Mean values and standard deviations)

Covariates	Vegetable consumption (portions/d)			Fruit consumption (portions/d)			F&V consumption (portions/d)		
	Mean	sd	*P*	Mean	sd	*P*	Mean	sd	*P*
Sex									
Male (*n* 14 718)	0⋅99	0⋅69	<0⋅001*	0⋅94	0⋅76	<0⋅001*	1⋅93	1⋅21	<0⋅001*
Female (*n* 20 589)	1⋅30	0⋅79		1⋅41	0⋅96		2⋅70	1⋅48	
Age (years)									
25–44 (*n* 11 168)	1⋅24	0⋅79	<0⋅001†	1⋅18	0⋅90	<0⋅001†	2⋅42	1⋅44	<0⋅001†
45–64 (*n* 16 115)	1⋅20	0⋅77		1⋅23	0⋅92		2⋅42	1⋅44	
65–84 (*n* 8024)	1⋅02	0⋅69		1⋅23	0⋅89		2⋅25	1⋅37	
Education level									
Basic (*n* 3325)	0⋅91	0⋅71	<0⋅001†	1⋅06	0⋅88	<0⋅001†	1⋅97	1⋅37	<0⋅001†
Secondary (*n* 13 296)	1⋅07	0⋅76		1⋅11	0⋅89		2⋅19	1⋅40	
Higher (*n* 18 686)	1⋅29	0⋅75		1⋅31	0⋅91		2⋅59	1⋅42	
BMI (kg/m^2^) in 2010									
BMI < 25⋅0 (*n* 19 489)	1⋅23	0⋅76	<0⋅001†	1⋅27	0⋅92	<0⋅001†	2⋅50	1⋅43	<0⋅001†
25⋅0 ≤ BMI < 30⋅0 (*n* 12 300)	1⋅10	0⋅75		1⋅13	0⋅89		2⋅23	1⋅39	
BMI ≥ 30⋅0 (*n* 3518)	1⋅07	0⋅77		1⋅10	0⋅91		2⋅16	1⋅44	
Total (*n* 35 307)	1⋅17	0⋅76		1⋅21	0⋅91		2⋅37	1⋅43	

* Mann–Whitney *U* test.

† Kruskal–Wallis test.

### Associations between fruit and vegetable intake and the onset of type 2 diabetes

The unadjusted model revealed that men who had fewer than two servings of vegetables per d were at a significantly higher at risk of developing T2D compared with those whose intake was two or more servings per d (model 1 unadjusted: OR 2⋅08; 95 % CI 1⋅29, 3⋅36). This association remained significant even after the adjustment for weight status in model 2 (OR 1⋅89; 95 % CI 1⋅17, 3⋅05); in model 3 adjusted for age and education (OR 1⋅78; 95 % CI 1⋅10, 2⋅89); in model 4 adjusted for age, education and weight status (OR 1⋅68; 95 % CI 1⋅04, 2⋅74); and in fully adjusted model 5 (OR 1⋅62; 95 % CI 1⋅00, 2⋅63). The strength of the association between vegetable consumption and the development of T2D in men was reduced by 9⋅0, 14⋅4, 19⋅0 and 22⋅0 % due to the confounding effects in model 2, model 3, model 4 and model 5, respectively, relative to the unadjusted model 1. Women who consumed fewer than two servings of vegetables per d had significantly higher risk (OR 1⋅57; 95 % CI 1⋅10, 2⋅24) for the development of T2D during this period than those who consumed two or more servings per d only in the unadjusted model. Furthermore, fruit and combined fruit and vegetable consumption was not associated with the development of T2D either in men or in women (Table 3). Moreover, we tested if there was an interaction effect between low vegetable intake and high BMI, which both were significant predictors of T2D risk. No significant interaction effect was found on T2D risk.

**Table 3.** Association between fruit and vegetable intake and new cases of type 2 diabetes (T2D) among men and women based on logistic regression (*n* 35 307) (Odds ratios and 95 % confidence intervals)

	T2D new cases									
	Model 1‡		Model 2§		Model 3||		Model 4¶		Model 5‡‡	
	OR	95 % CI	OR	95 % CI	OR	95 % CI	OR	95 % CI	OR	95 % CI
Men (*n* 14 718)										
Vegetable intake										
≥2 servings/d (ref.)	1·00		1·00		1·00		1·00		1·00	
<2 servings/d	2·08**	1·29, 3·36	1·89**	1·17, 3·05	1·78*	1·10, 2·89	1·68*	1·04, 2·74	1·62†	1·00, 2·61
Fruit intake										
≥2 servings/d (ref.)	1·00	0·74, 1·70	1·00	0·68, 1·56	1·00	0·74, 1·69	1·00	0·70, 1·61	1·00	0·69, 1·59
<2 servings/d	1·13		1·03		1·12		1·06		1·04	
F&V intake										
≥4 servings/d (ref.)	1·00	0·77, 2·40	1·00	0·69, 2·17	1·00	0·72, 2·26	1·00	0·68, 2·14	1·00	0·66, 2·08
<4 servings/d	1·36		1·22		1·28		1·20		1·17	
Women (*n* 20 589)										
Vegetable intake										
≥2 servings/d (ref.)	1·00	1·10, 2·24	1·00	1·00, 2·06	1·00	0·83, 1·72	1·00	0·83, 1·72	1·00	0·82, 1·71
<2 servings/d	1·57*		1·44†		1·19		1·19		1·18	
Fruit intake										
≥2 servings/d (ref.)	1·00	0·71, 1·36	1·00	0·66, 1·28	1·00	0·67, 1·28	1·00	0·66, 1·27	1·00	0·65, 1·26
<2 servings/d	0·98		0·92		0·92		0·91		0·90	
F&V intake										
≥4 servings/d (ref.)	1·00	0·87, 1·86	1·00	0·79, 1·70	1·00	0·75, 1·60	1·00	0·73, 1·58	1·00	0·73, 1·58
<4 servings/d	1·27		1·26		1·09		1·08		1·07	

ref., Reference group; F&V, fruit and vegetables.

Indicates level of significance: * *P* < 0·05, ** *P* < 0·01.

† Borderline significant (*P* = 0·05).

‡ Unadjusted.

§ Adjusted for weight status.

|| Adjusted for age (continuous) and education (categorical).

¶ Adjusted for age, education and weight status (categorical).

‡‡ Adjusted for age, education, weight status, smoking, alcohol and physical activity.

### The goodness-of-fit test

The Hosmer and Lemeshow test, *P* > 0⋅05, and the area under the ROC curve, >0⋅70, confirmed that the estimated models predicted the outcome better compared with the unadjusted model (data not shown).

### Sensitivity analysis

The sensitivity analysis comparing the results between individuals with complete data (Table 3) and all participants (Table 4) demonstrated no major difference in results due to lack of information on covariates. The level of significance of the associations between fruit and vegetable intake and T2D risk did not change in the larger sample size compared with the smaller sample with complete data on all covariates.

**Table 4.** Association between fruits, vegetables, and fruit and vegetable (F&V) consumption and the onset of type 2 diabetes (T2D) among Swedish adults, based on logistic regression in the total sample (*n* 39 914) (Odds ratios and 95 % confidence intervals)

	T2D new cases							
	Men (*n* 16 624)				Women (*n* 23 290)			
	Model 1†		Model 2‡		Model 1†		Model 2‡	
	OR	95 % CI	OR	95 % CI	OR	95 % CI	OR	95 % CI
Vegetable intake								
≥2 servings/d (ref.)	1·00		1·00		1·00		1·00	
<2 servings/d	2·16**	1·39, 3·37	1·92*	1·23, 3·00	1·57*	1·13, 2·18	1·33	0·95, 1·85
Fruit intake								
≥2 servings/d (ref.)	1·00		1·00		1·00		1·00	
<2 servings/d	1·23	0·84, 1·81	1·25	0·85, 1·84	1·01	0·75, 1·36	1·01	0·75, 1·37
F&V intake								
≥4 servings/d (ref.)	1·00		1·00		1·00		1·00	
<4 servings/d	1·71	0·97, 3·00	1·66	0·94, 2·92	1·33	0·94, 1·89	1·25	0·88, 1·78

ref., Reference group.

Indicates level of significance: * *P* < 0·05, ** *P* < 0·01.

† Unadjusted.

‡ Adjusted for age (continuous).

## Discussion

### Main findings and interpretation

The study aspired to examine the risk of a low intake of fruit and vegetables for future development of T2D in men and women aged 25 to 84 years living in Stockholm County during the period 2010–2014.

The main finding was that a low intake of vegetables was associated with a significant risk of developing T2D during a 4-year follow-up period in men, but not in women. Only 21 % of men and 37 % of women consumed two or more servings of vegetables per d. As expected, the relative risk decreased considerably in both men and women when adjusting for the confounders age, education, weight status, smoking, alcohol consumption and physical activity. Our findings are compatible with the idea of a safe threshold level of two to three servings of vegetables per d^(14,15)^. There are several possible explanations for the differential findings in men and women concerning vegetable intake and risk of T2D. First, fewer women than men were found in the low intake category, which reduces the power of the analysis in women. This could explain the weaker association in women, which did not reach statistical significance after adjustment for covariates. Another explanation could be that men in general prefer other types of vegetables than women. A meta-analysis suggested that only root or green leafy vegetables were associated with lower risk of T2D^(33)^. More research is needed to answer this question with detailed assessment of which specific vegetables are consumed by men and women, respectively. Finally, we checked that the differential risk among men and women could have been due to the combined presence of synergistic risk factors such as high BMI (55⋅1 *v*. 37⋅5 % with BMI ≥ 25⋅0 kg/m^2^ in men *v*. women), and low vegetable intake (79⋅0 *v*. 62⋅6 % with fewer than two servings/d in men *v*. women) which was more prevalent in men than in women in our study (Table 1). However, the interaction analysis did not suggest such synergistic effect (results not shown). The small but significant decrease in the average intake of fruit and vegetables from 2010 to 2014 was surprising, because the general consciousness of the health effects of fruit and vegetables in the Swedish population had most probably not decreased during these years.

### Fruits and risk of type 2 diabetes

In contrast to results found in four meta-analyses^(14,15,34,35)^, we did not find a reduced T2D risk associated with a higher intake of fruit, neither in men nor women. Our finding is in line with the European Prospective Investigation into Cancer and Nutrition-Norfolk study, where Cooper *et al*.^(36)^ observed that a higher intake of vegetables but not fruits was associated with lower risk of T2D and with Kurotani *et al*.^(37)^ who found that neither intake of fruits nor fruit and vegetables combined was associated with T2D risk. We can only speculate about the reason for this discrepancy concerning the role of fruit in the development of T2D across different studies. As with vegetables, there is the possibility that certain fruits but not others offer protection against the disease, and that different populations have different preferences and availability of fruits. A recent review of mechanisms of T2D risk reduction suggests that polyphenols, and specifically flavonoid compounds, may have an important role in preventing or delaying the onset of T2D^(38)^. The dietary intake of these compounds varies five-fold among different regions of the world^(39)^. Foods rich in flavonoids such as anthocyanins found in, for example, strawberry, blackberry, bilberry, blackcurrant or pomegranate and blueberries, and flavan-3-ols found in, for example, plum, apple, custard apple, strawberry-tree fruit, blueberry and cranberry, cherry and grapes may confer specific benefits through pathways influencing glucose absorption and insulin sensitivity and/or secretion^(38)^. Previous studies suggest that fibre from fruit and vegetables does not seem to be involved in the protective effects against T2D^(40)^. Thus, much more detailed dietary assessment or alternatively studies using biomarkers will be required to clarify why studies in some populations show protective effects of fruit on T2D risk and not in others.

### Strengths and limitations

This study has several strengths. First, it has a longitudinal design, which in combination with measurement of confounders and health at baseline provides a higher certainty to interpret the results causally. Second, intake of fruit and vegetables was assessed both at baseline and at follow-up, showing a relatively stable level of intake during the 4 years. Third, as the study involves a large prospective population cohort, there is a reduced risk of reverse causation and selection bias^(41)^. Fourth, the response rate at follow-up was relatively high, which provides high statistical power. Finally, since all participants live in the same region (Stockholm County), there were no differences in potential macro-environmental confounders such as climate, environmental contamination and access to health facilities^(5)^.

This study also has some limitations. First, the follow-up time is only 4 years, which is a relatively short period compared with other studies. Furthermore, information on disease onset was by self-report based on the self-reported age at diagnosis. It would have been better to obtain this information from registries, although we think that most individuals would report their health condition truthfully. Self-report of dietary intake by questionnaire is a crude method and constitutes a well-known weakness due to recall bias and social desirability bias, which means that there is an obvious risk of misclassification of the number of portions of fruit and vegetables consumed. For the same reason we chose to have only two intake categories (above or below current recommendations). We tested the possibility of using three intake categories, but this did not change the results (results not shown). In order to obtain more precise risk estimates regarding fruit and vegetable intake one would have to design a study specifically for this purpose. Third, data were collected only for two time points, and therefore mediation analysis was not possible to perform. Moreover, even though it is a large prospective cohort, some selection bias was seen in that participants were to a higher extent female and with higher education. However, this is not expected to affect the risk estimates associated with intake of fruit and vegetables. Fourth, subsequent adjusted models (Table 3) suggested that men, not women, who had a low level of intake of vegetables were significantly more likely to develop T2D; however, we analysed the effect modification of sex on vegetable intake and development of T2D, and there was none.

The interpretation regarding causality between vegetable intake and T2D should nevertheless be done with great caution, as one can only infer causality with certainty from randomised controlled trials^(42)^. Moreover, a misclassification between T2D and latent autoimmune diabetes of adults^(43)^, and of type 1 diabetes as T2D and vice-versa might have occurred. One major problem is the high rate of undiagnosed disease as about one-third of all diabetics have not yet received a diagnosis^(19)^. This could have led to outcome misclassification and lower risk estimates than in reality. Although we adjusted for several confounders, there might have been residual confounding by unmeasured dietary and other factors associated with a low vegetable intake which might have affected the results.

### Future research

While the protective role of vegetables against T2D was confirmed by our study, the role of fruit in T2D development is still in question and needs more research. Furthermore, it seems important to investigate if specific types of fruit and vegetables are more protective than others by investigating mechanisms of action. To do that a food diary and ideally blood samples would be required in order to analyse nutritional biomarkers of intake^(44)^. Future research should also aim at developing and evaluating interventions which will increase the intake of fruit and vegetables in the population, and especially among men. Sex differences in fruit and vegetable intake are already notable in children^(45)^, and interventions should therefore start in early childhood.

### Conclusions

In conclusion, the results of our study suggest that the overall mean consumption of fruit and vegetables in Stockholm County is low compared with the recommended level of consumption, and slightly lower than in some other countries in the European Union. In men, higher consumption of vegetables was found to be protective against the onset of T2D after adjusting for age, education, BMI, smoking, alcohol and physical activity, but not as clearly in women. Fewer women were low consumers of fruit and vegetables than men, and risk estimates for women might therefore have had lower statistical power. Fruit consumption was not associated with T2D risk neither in men nor women. In order to clarify the mechanisms behind these differential effects of fruit and vegetables more research is needed with more detailed assessment of intake of specific fruits and vegetables.
